# Complete diploid genome of the type strain *Yarrowia lipolytica* YB-423 (ATCC 18942™)

**DOI:** 10.1093/jimb/kuag002

**Published:** 2026-01-08

**Authors:** James E Crill, Scott V Nguyen, Corina Tabron, Nikhita Puthuveetil, Anthony Muhle, Zethus W Avery, Joseph Petrone, Kaitlyn Bentley, Jade Kirkland, Noah Wax, Robert Marlow, James Duncan, Steve King, Ana Fernandes, John Bagnoli, Briana Benton, Shahin S Ali, Roy D Welch, Jonathan L Jacobs

**Affiliations:** Forensic & National Security Sciences Institute (FNSSI), Syracuse University, Syracuse, NY 13244, United States; Department of Biology, Syracuse University, Syracuse NY 13244, United States; ATCC, Manassas, VA 20110, United States; ATCC, Manassas, VA 20110, United States; ATCC, Manassas, VA 20110, United States; ATCC, Manassas, VA 20110, United States; Forensic & National Security Sciences Institute (FNSSI), Syracuse University, Syracuse, NY 13244, United States; ATCC, Manassas, VA 20110, United States; ATCC, Manassas, VA 20110, United States; ATCC, Manassas, VA 20110, United States; ATCC, Manassas, VA 20110, United States; ATCC, Manassas, VA 20110, United States; ATCC, Manassas, VA 20110, United States; ATCC, Manassas, VA 20110, United States; ATCC, Manassas, VA 20110, United States; ATCC, Manassas, VA 20110, United States; ATCC, Manassas, VA 20110, United States; ATCC, Manassas, VA 20110, United States; Department of Biology, Syracuse University, Syracuse NY 13244, United States; Forensic & National Security Sciences Institute (FNSSI), Syracuse University, Syracuse, NY 13244, United States; Forensic & National Security Sciences Institute (FNSSI), Syracuse University, Syracuse, NY 13244, United States; ATCC, Manassas, VA 20110, United States

**Keywords:** Yeast genetics, bioinformatics, type-strain, genome assembly, genome annotation

## Abstract

Here, we present the first complete, fully phased diploid genome of type strain *Yarrowia lipolytica* YB-423 (=ATCC 18942™), constructed using a combination of Oxford Nanopore long-read and Illumina short-read sequencing. *Yarrowia lipolytica* is an industrially relevant yeast species known for its metabolic versatility, particularly its ability to degrade hydrophobic compounds and express useful products such as fatty acids. Despite its growing use in biotechnology, a high-quality genome assembly of the species’ diploid type-strain has been lacking. The assembly and annotations presented here span six chromosomes of paired “haplotigs” and a mitochondrial genome, capturing large-scale structural variations and prominent levels of genome-wide heterozygosity. Variant analysis revealed 13,908 heterozygous alleles, of which 3,201 alleles were distributed among 1,237 protein-coding genes. Gene set enrichment analysis showed that these variants are enriched among genes involved in transmembrane transport, suggesting a role in environmental adaptability. Comparative analysis of matched haplotigs for the same chromosome uncovered multiple inversions and transpositions, as well as allele-specific insertions of retrotransposons, providing new insights into the structural complexity and evolutionary dynamics of the genome. The fully phased, finished diploid genome of *Y. lipolytica* YB-423 represents a crucial step toward unlocking the full genetic potential of *Y. lipolytica*. Our work will provide a valuable foundation for future comparative and functional genomics and strain engineering studies, particularly for industrial microbiology and biotechnology applications.

**One-sentence summary** This study presents the first fully phased diploid genome of *Yarrowia lipolytica* type strain YB-423, revealing extensive structural variation and heterozygosity that enhance understanding of its genetic adaptability and industrial potential.

## Graphical Abstract

**Graphical Abstract:**
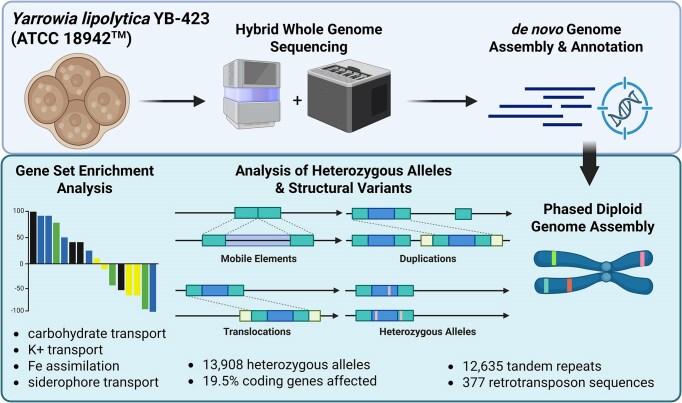

## Significance

This study provides the first fully phased diploid genome assembly of the type-strain for *Yarrowia lipolytica*. Using a combination of short-read and long-read sequencing, the authors produced a complete genome that clarifies structural features that were unresolved in previous assemblies. The new genome reveals over 13,000 heterozygous variants, including functional changes in genes linked to membrane transport. These findings are important for researchers and industry professionals interested in microbial genetics, biotechnology, and environmental applications. This new reference genome offers a robust platform for strain engineering, particularly for improving metabolic pathways. Both the haploid consensus and phased diploid assembly for *Y. lipolytica* YB-423 are available from the ATCC Genome Portal (https://genomes.atcc.org).

## Introduction

*Yarrowia lipolytica* is a versatile, oleaginous yeast with a high potential for broad biotechnology applications. This non-pathogenic yeast, belonging to the *Ascomycota* phylum, has garnered attention not only for its unique morphological plasticity—fluctuating between yeast-like cells, pseudohyphae, and true hyphae based on environmental cues—but also for its robust genetic and biochemical characteristics that enable a myriad of biotechnological applications (Park & Ledesma-Amaro, 2023; Zieniuk et al., 2024).

Briefly, *Y. lipolytica* was likely first identified in the early 20th century by Lore A. Rogers (Jordan, 1903) as a “fat-splitting torula” responsible for butter spoilage. Rogers later published his characterization of this unusual yeast (Rogers, 1904), and soon after other researchers found it to be a common contaminant of many high-fat food products, such as vegetable margarine (Harrison, 1928; Jacobsen, 1919). Efforts to characterize its “fat-splitting” enzymatic capabilities led to the discovery and characterization of novel lipases for use in the food industry (Long, 1936; Peters & Nelson, 1948); these same enzymes were also found to be capable of metabolizing a wide range of hydrocarbons (Davis, 1956). Proponents of “single-cell protein” production in the 1960s suggested using industrial petroleum-based waste products as feedstocks for microorganisms such as *Y. lipolytica*. Early studies met substantial success, which in turn led to dozens of patents and the eventual commercialization of several alternative foods produced from *Y. lipolytica* cultured on petrochemical wastes (Bamberg, 2000; Hiroshi Iizuka, 1965; Johnson, 1967; Weyn HF, 1977; Wickerham & Kuehner, 1956). These advancements in industrial microbiology also drove fundamental discoveries in yeast genetics. Importantly, the creation of hybrid strains of *Y. lipolytica* (Wickerham et al., 1970a) allowed for the selection of strains with distinct phenotypic advantages for various industrial applications.

The earliest known isolate for the species, however, was deposited to the Centraalbureau voor Schimmelcultures (CBS) collection in 1921, now known as *Y. lipolytica* CBS 599 (=CCRC 20864; =DBVPG 6132; =IFO 1195; =VKM Y-47). CBS 599 is a haploid strain originally described as *Mycotorula lipolytica* and was isolated in 1919 from contaminated margarine by HC Jacobsen at the Antwerp Jurgens Margarine Works—the first industrial scale margarine manufacturing plant (Jacobsen, 1919; Kluyver, 1952). In 1928, FC Harrison formally described the species for the first time as *M. lipolytica* Jacobsen (Harrison, 1928), but the specific strain he described was unfortunately not accessioned into any culture collection.

The type strain of *Y. lipolytica* (=NRRL YB-423^T^; =ATCC 18492^T^; =CBS 6124^T^; =DBVPG 6053^T^; =IFO 1548^T^; =JCM 2320^T^) is a wild-type diploid, ascosporogenous yeast originally isolated from fiber tailings at a corn processing plant in Illinois by USDA microbiologist LJ Wickerham in 1945 (Wickerham et al., 1970b). Wickerham’s formal description and designation of the type material for the species (YB-423) came decades later, and placed it into the newly assigned genus *Endomycopsis lipolytica* (Wickerham et al., 1970b). The same year, the type strain was accessioned into the American Type Culture Collection (now ATCC) under catalog number ATCC 18942 in response to increasing research interest in its mating types and its use in generating bisexual cultures (Wickerham, 1970, ARS Letter to ATCC). Subsequent nomenclatural harmonization efforts by David Yarrow in 1972 consolidated multiple synonyms for the species under *Saccharomycopsis lipolytica* (Yarrow, 1972). The species was, however, reassigned again to the genus *Yarrowia* by JP van der Walt and JA von Arx, honoring Yarrow’s life-long contribution to the field of mycology (van der Walt & von Arx, 1980).

The first draft whole-genome assembly for *Y. lipolytica* (GCF_000002525.2) was produced from strain CLIB 122 (=E150), a haploid genetically inbred strain derived from an initial cross of W29 (=ATCC 20460™; =CLIB 89) and YB-423–12 (=ATCC 18944™; =CLIB 78). The initial progeny was then serially backcrossed to W29 (Dujon et al., 2004; Kerscher et al., 2001). YB-423–12 is the direct haploid progeny of the type strain YB-423 (=ATCC 18942), thus making CLIB 122 a genetic descendant of the type strain. In 2015, the assembly for CLIB 122 was suppressed in the NCBI RefSeq database and replaced by a new reference finished genome assembly based on W29 (Pomraning & Baker, 2015).

Fully phased, diploid genome assemblies for eukaryotic microbes remain rare in public databases, a reflection of the challenges faced in creating them given the limitations of available data and technologies. The first complete yeast genome was haploid and completed in 1996, after a considerable international effort from dozens of laboratories (Goffeau et al., 1996). Even as sequencing technologies continued to advance, the first phased diploid genome assembly for a eukaryotic microbe was not released until 2013 for *Candida albicans* (Muzzey et al., 2013), and largely relied on short-read sequencing data mapped to a consensus reference genome built from dozens of haploid assemblies. It was not until 2022, that a fully phased, chromosome scale genome assembly for a diploid eukaryotic microbe first became available, namely the wheat leaf rust fungus *Puccinia triticina* (Duan et al., 2022). Since then, phased diploid genome assemblies for yeasts and other fungi remain rare occurrences. Currently, of the 53 draft genome assemblies for *Y. lipolytica* in GenBank, only one is from a diploid strain: a fragmented draft assembly (GCA_030581655.1) comprising 426 contigs for type strain YB-423 (Opulente et al., 2024). Unfortunately, this assembly has been erroneously mislabeled as for a haploid strain in GenBank. As far as the authors of this work are aware, only three diploid strains for *Y. lipolytica* have ever been sequenced, assembled and annotated: YB-423, the type-strain as presented here in this work; DX271 (=ATCC 46069™), an inbred derivative of type-strain descendants (Simms & Ogrydziak, 1981); and D.1805 (=ATCC 20390™), a previously patented laboratory strain developed for citric acid production (Briffaud & Engasser, 1979). All three have been fully sequenced by ATCC and the corresponding data is available from the ATCC Genome Portal (Nguyen et al., 2024).

Given the necessity of establishing high-quality reference genomes for microbial type strains, here, we present our results of creating a complete, fully phased reference genome for *Y. lipolytica* YB-423 (=ATCC 18942). Our results identify thousands of heterozygous variations present in the type strain genome that would otherwise have been masked in a haploid consensus assembly, including single-nucleotide variants (SNVs), insertions/deletions (Indels), and larger mobile elements such as Ty3 and Ty6 transposons. This new genome assembly provides an improved foundation for our understanding of *Y. lipolytica* genetics that will enable improvements in producing genetically modified derivative strains for future industrial applications and serve as a standardized reference for future comparative genomics studies.

## Materials and methods

### Culturing and sample preparation

*Yarrowia lipolytica* YB-423 (=ATCC 18942) was cultured in Yeast Mold Broth (BD-271120, AT, Becton Dickinson, Franklin Lake, NJ, USA) at 28 °C for 48 hr. Cells were harvested, washed twice with phosphate buffered saline, and dried using vacuum centrifugation. For the haploid consensus assembly, genomic DNA was extracted from 0.2 g of the dried cell pellet using the Zymo *Quick*-DNA Fungal/Bacteria Miniprep Kit (Zymo Research Corporation, Irvine, CA, USA). DNA quality and concentration were assessed using a NanoDrop spectrophotometer and Qubit fluorometer, respectively (Thermo Fisher Scientific, Waltham, MA, USA). The final DNA preparation yielded 26.3 ng/µl in 100 µl of elution buffer, with 260/230 and 260/280 absorbance ratios of 2.182 and 1.846, respectively.

### Whole-genome sequencing

#### Illumina sequencing

Paired-end sequencing libraries were prepared from half of the extracted DNA using an Illumina (ILMN) DNA Prep Kit (Illumina Corporation, San Diego, CA, USA) and indexed using ILMN’s DNA/RNA unique dual (UD) indexes. Subsequent sequencing was conducted on an ILMN NextSeq 2000 instrument.

#### Oxford nanopore technologies sequencing

Long-read sequencing was performed with DNA from the same extraction described above for ILMN sequencing using an Oxford Nanopore Technologies (ONT) Ligation Sequencing Kit (Oxford Nanopore, UK, SQK-LSK109). ONT libraries were indexed and multiplexed using the Native Barcoding Expansion Kit (EXP-NBD104 or EXP-NBD114), and sequencing was performed on a GridION using both the MinION Flow Cell R9.4.1 and R10.4.1 chemistries. Libraries were sequenced for 72 hr.

#### Haploid genome assembly

Following ILMN base-calling and adapter trimming, an additional round of trimming and quality-score filtering was carried out using *fastp* (S. Chen et al., 2018) with the following thresholds: median Q score >30; median Q score, per base >25; and ambiguous content (% N bases) <5%. For ONT reads, sequences were base-called using ONT’s cloud-based *MinKNOW* service using the high accuracy settings, demultiplex, and barcode trimming. Subsequent quality trimming and filtering was carried out using *filtlong* (De Coster et al., 2018) to meet the following minimum acceptance criteria: minimum mean Q score per read >10 and minimum read length >1,000 bp.

The type strain genome size was estimated from ILMN raw reads using *mash* (Ondov et al., 2016), and this estimate was used to down-sample both the ILMN- and ONT-trimmed sequencing libraries to an estimated 100× and 40× coverage, respectively. Following down-sampling, hybrid *de novo* assembly was carried out using the *MaSuRCA* pipeline (Zimin et al., 2013) with *flye* assembler (Kolmogorov et al., 2019) using the ONT and ILMN reads. Contigs and assembly artifacts of less than 1,000 bp that also had significantly different coverage depth when compared to the genome-wide median (e.g., “chaff” contigs) were removed from the final draft reference. These draft assemblies were subsequently screened using *kraken2* (Wood et al., 2019) for species confirmation and contamination screening. The draft assembly was then polished with the ILMN reads using *polypolish* (Wick & Holt, 2022). The draft assembly was checked for completeness with *BUSCO* (Manni et al., 2021).

#### Diploid genome assembly

Assembly of a phased, diploid genome utilized the following approach. First, *HERRO v1* (Stanojevic et al., 2024), as implemented in *dorado* v0.8.1 (https://github.com/nanoporetech/dorado), was used to generate error-corrected long reads from the high-molecular-weight DNA described above. A *de novo* assembler, *necat* v0.0.1_update20200803 (Y. Chen et al., 2021), was next used for assembling YB-423 using the *HERRO*-corrected ONT reads with default parameters with the following exceptions: prep_output_coverage = 80, cns_output_coverage = 100. From the initial *necat* assembly, a phased, diploid assembly was produced using *hapdup* v0.12 (Kolmogorov et al., 2023) with the prior *HERRO* corrected reads. Next, polishing was done with both *racon* (Vaser et al., 2017) and *minipolish* (Wick & Holt, 2019) using 10 M ILMN reads randomly subsampled from the initial hybrid-assembly. Lastly, the two mitochondrial haplotig sequences were merged to produce a single consensus mtDNA reference using *CLC Genomics Workbench and Server v24* (CLC, QIAGEN, Aarhus, Denmark). Genome annotation of each set of haplotigs followed the approach described above.

#### Genome annotation

Annotation of the haploid consensus genome assembly was conducted using *CLC*. Briefly, 25 pre-existing genome assemblies for *Y. lipolytica* were downloaded from NCBI. All annotated CDS, rRNA, tRNA, ncRNA, and mobile elements were extracted from these assemblies, deduplicated, and then used as inputs to annotate the type strain draft assembly using BLAST (Altschul et al., 1990). Furthermore, gene ontology (GO) assignments and further refinement of start and stop codon positions were made for CDS regions using CLC’s implementation of *DIAMOND* (Buchfink et al., 2015) with *PFAM* R64 (Mistry et al., 2021). Tandem repeat analysis was conducted with *Tandem Repeat Finder* v4.10.0 (Benson, 1999) with the following parameters: match 2, mismatch 5, indel penalty 7, match probability 80, indel probability 10, minimum score 50, and maximum period 2,000.

#### Heterozygosity analysis

For heterozygous variant allele detection, 57.5 M reads from two independent ILMN library preps were mapped back to the haploid assembly with CLC’s read mapper using default settings. Variant calling was performed with CLC’s implementation of *LoFreq* (Wilm et al., 2012) using the default settings and a *p* < 0.01 significance cutoff. Additional filtering of the variants used the following criteria: minimum frequency 25%, minimum forward/reverse read balance of 5%, minimum depth of coverage for the allele 80×, average base quality ≥Q30, and baseqranksum ≥−1.65. Furthermore, alleles not found in predicted homopolymer regions or repeat regions were excluded from further variant analysis. For regions with unexpectedly high coverage, read mapping coverage fitted to a Poisson distribution and regions of at least 50 bp with a *p*-value <0.0001 were annotated as being high coverage regions.

#### Comparative genomics

Comparative genomic whole-genome alignments (WGA) were performed using CLC’s implementation of *progressiveMauve* (Darling et al., 2010) with a modified HOXD substitution matrix (Chiaromonte et al., 2001) using the following parameters: minimum initial seed length = 9, mismatches allowed, and minimum locally colinear block (LCB) length = 36. Analysis of retrotransposons was carried out using alignment-free k-mer spectra analysis (k-mer size 15, NJ tree construction) for initial clustering (Höhl et al., 2006). Individual groups of clustered sequences were then further aligned using *Clustal W* (Larkin et al., 2007) and visualized in CLC.

## Results

**Figure 1:**
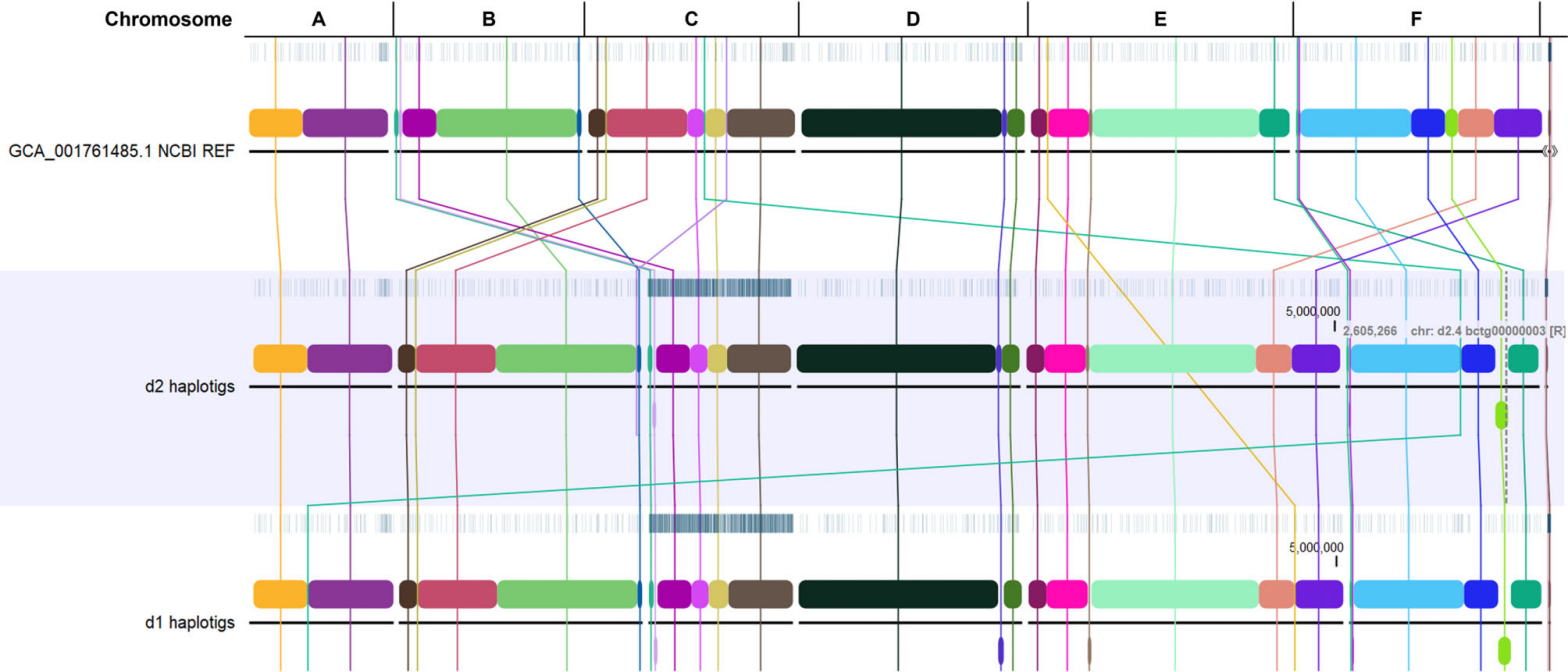
WGA of each set of haplotigs from the diploid assembly of *Y. lipolytica* YB-423 (=ATCC 18942) against the NCBI RefSeq assembly for strain CLIB89/W29 with respective chromosome names indicated. Local colinear blocks (LCBs) are shown as colored boxes with their homologous blocks in each assembly connected by identically colored lines.

In the results and discussion that follows, we present two distinct assemblies produced from the same originating data for the type strain: a haploid consensus assembly, which represents our initial approach to hybrid *de novo* assembly using *MaSuRCA* and *flye* assemblers with ONT and ILMN reads, followed by genome annotation and variant calling; and a phased diploid assembly consisting of two sets of sister “haplotigs” (d1 and d2) from the diploid assembly, each represented by six pairs of chromosomes produced via *de novo* assembly using *necat* in conjunction with *hapdup* (see Methods). For comparison purposes, we carried out WGA of each set of haplotigs against the RefSeq assembly for the haploid W29 (=ATCC 20460™) strain (Figure 1 below).

### Haploid assembly analysis

The initial haploid consensus assembly resulted in a scaffold-level assembly of 34 contigs 20.9 MB in length (N50: 2,014,421 bp, GC content: 49%), in line with a genome-size estimation made using *mash* (Ondov et al., 2016) prior to assembly. Although this was a significant improvement over the prior draft assembly of 426 contigs (Opulente et al., 2024), our analysis shows significant heterozygosity at both the allelic and structural genome levels, and further improvements in a haploid consensus assembly were not likely possible. Nonetheless, genome completeness for this assembly was evaluated using *BUSCO* (Manni et al., 2021), which identified 743 core genes, consistent with other complete *Y. lipolytica* genomes available from the ATCC Genome Portal (Nguyen et al., 2024) and NCBI GenBank (Goldfarb et al., 2024). Genome annotation of the assembly resulted in 6,876 genes, comprising 6,352 protein-coding genes, 524 tRNAs, 137 rRNAs, 28 putative ncRNA genes, and 88 potential mobile elements.

To assess genome-wide heterozygosity, we mapped ILMNreads from two independent libraries produced from the same starting material back to the haploid consensus assembly described above. Over 99% of reads mapped successfully with a median 361× coverage. Variant calling revealed 15,305 heterozygous alleles, reduced to 13,908 high quality, non-homopolymer variants following initial filtering (see Materials and methods). These heterozygous variants included 12,855 SNVs, 214 multi-nucleotide variants (MNVs), 504 insertions, 264 deletions, and 71 replacement variants. Many of these variants (10,707) were found in intergenic regions. A total of 3,201 heterozygous variants occurred within genes, including 2,139 synonymous and 1,062 non-synonymous variants affecting collectively 1,237 protein-coding genes (~19.5% of all CDS regions). In addition, SNVs located within 2 bp of exon-intron junctions were detected in seven genes, which may potentially have a role in allelic alternative splicing and downstream protein expression. All variants and their associated data are detailed in Table S1: Heterozygous Variants.

### Gene set enrichment analysis of heterozygous alleles

Gene set enrichment analysis (GSEA) was performed on the haploid consensus assembly to investigate whether specific biological pathways harbored heterozygous variants more than otherwise expected (Falcon & Gentleman, 2007). Using existing GO annotations for *Y. lipolytica* W29 (GCF_001761485.1), a total of 4,161 genes were assigned to GO biological process. Collectively, 2,135 heterozygous variants were found among 763 genes with an assigned GO term, and from which 535 non-synonymous variants were found among 349 genes (Table S2: GO Variants). GSEA identified 60 significantly enriched GO terms (*p* ≤ 0.05) among genes harboring heterozygous variants (Table S3: GSEA).

Interestingly, “transmembrane transport” (GO: 0055085) was the most significantly enriched for genetic heterozygosity (80 out of 302 genes, *p* = 1.99E-04). In addition, among the top 20 enriched biological processes, terms associated with carbohydrate transmembrane transport (GO: 3219), carbohydrate transport (GO: 8643), potassium ion transmembrane transport (GO: 71805), iron assimilation by chelation and transport (GO: 33214), and siderophore transmembrane transport (GO: 44718) were also enriched. An enrichment of heterozygous alleles associated with iron and carbohydrate metabolism and transport may be particularly relevant given recent evidence specifically associating iron availability with *Y. lipolytica* lipid production (Cordova et al., 2022).

The functional consequences of these variants remain to be investigated; however, the overrepresentation of heterozygous variants in genes with roles in transmembrane transport or iron and carbon metabolism is intriguing given their roles in substrate uptake, efflux, and stress response—processes central to *Y. lipolytica’s* industrial utility. This observation raises the possibility that allelic diversity in these transporters may contribute to the strain’s metabolic adaptability for a diverse range of biotechnology applications, and thus warrants further investigation.

### Phased, diploid genome assembly

To provide chromosome-level haplotype resolution for the diploid type-strain, we produced a fully phased genome assembly for YB-423 (=ATCC 18942). To this end, we used an alternative assembly pipeline supplemented with additional ONT data. This new ONT data and the prior data were also re-basecalled using *dorado*, and the combined data were subjected to multiple reference-free ONT read error-corrections, ONT-only *de novo* assembly and scaffolding using *necat* (Y. Chen et al., 2021), and joint polishing of haplotigs with subsampled ILMN reads (see Materials and methods). Our approach yielded an assembly of six contiguous, fully finished chromosomal haplotig pairs (representing the nuclear genome) and a closed consensus genome for the mitochondrial DNA (mtDNA). An overview of all haplotigs and the mtDNA sequence is shown in Table 1. Following assembly of the diploid genome, ILMN and ONT reads were mapped back to the genome sequence and genome-wide structural comparisons of each pair of haplotigs (designated d1 and d2 for each chromosome set) were conducted using pairwise WGA. Notable structural differences emerged across each pair of haplotigs, as discussed below.

**Table 1:** Overview of phased, diploid assembly.

Chr	Haplotig	Length	Homologous W29Chr	Genes	CDS	rRNA	tRNA	ncRNA	TRR	Transposon
1	d1.1	5,105,959	E/F	2,209	1,888	27	128	5	1,026	35
1	d2.1	5,088,814	E/F	2,206	1,889	27	128	5	907	32
2	d1.2	3,949,622	B/C	1,538	1,284	27	82	7	1,201	35
2	d2.2	3,948,925	B/C	1,533	1,284	27	82	7	1,047	35
3	d1.3	3,628,337	D	1,440	1,209	16	63	5	1,305	25
3	d2.3	3,625,163	D	1,443	1,202	16	63	5	1,170	31
4	d1.4	3,129,913	E/F	1,272	1,083	18	39	4	1,118	19
4	d2.4	3,135,411	E/F	1,276	1,084	18	39	4	953	24
5	d1.5	2,339,438	B/C	979	767	13	71	1	1,005	24
5	d2.5	2,329,544	B/C	975	746	13	71	1	860	27
6	d1.6	2,339,498	A	1,080	760	36	137	4	1,072	48
6	d2.6	2,319,171	A	1,067	754	36	136	4	901	42
mtDNA	–	47,844	MT	45	16	2	24	0	35	0

Chr, chromosome labeling for this assembly. Haplotig, specific name for each haplotig in a chromosome paired set. Length, nt length. W29Chr, name of the equivalent chromosome from the W29 reference assembly, with recombination breaks indicated by a “/” slash. Genes, CDS, rRNA, tRNA, ncRNA, and Transposon indicated number of these elements found.

### Haplotig structural differences

**Figure 2:**
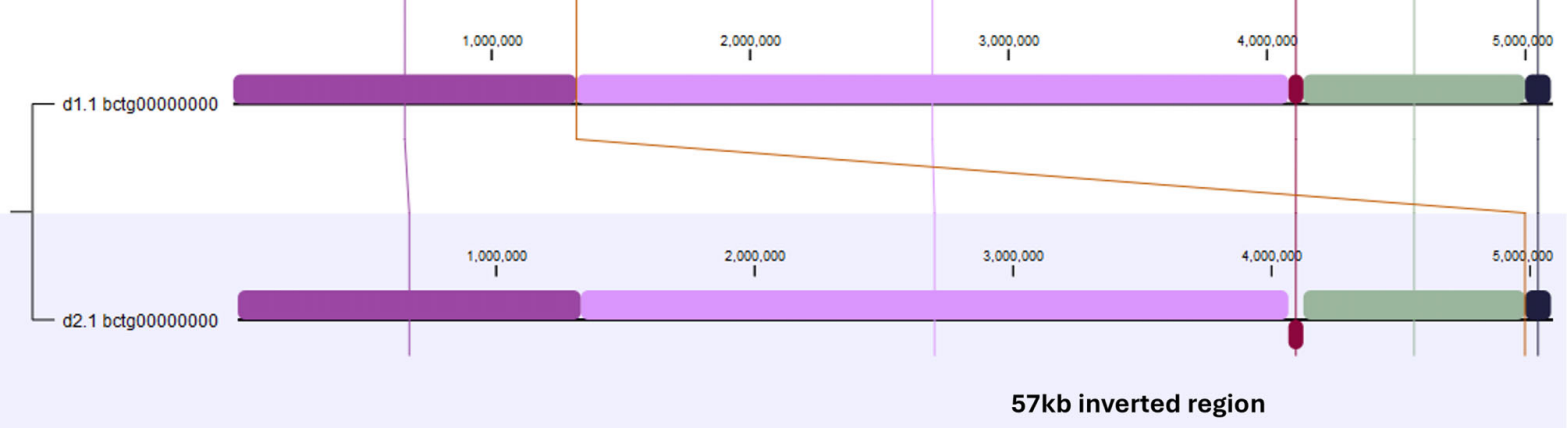
WGA of haplotigs d1.1 and d2.1 for chromosome 1 of *Y. lipolytica* YB-423 (ATCC 18942).

The haplotigs for chromosome 1 (haplotigs d1.1 and d2.1, Figure 2) differed in length by approximately 17 kb (5,105,959 bp vs. 5,088,814 bp). The size length was largely due to the presence of two Ylli, two Tyl3/Gyspy, and at least one additional uncharacterized retrotransposon being present in only one of the two haplotigs. In addition, we observed a significant 57.7 kb inversion comprising 18 protein-coding genes and a 5S rRNA gene (maroon LCB, Figure 2). In addition, d1.1 was found to contain a 4.2 kb Ylli transposon element not found in d2.1 and numerous additional smaller transposon relics.

**Figure 3:**
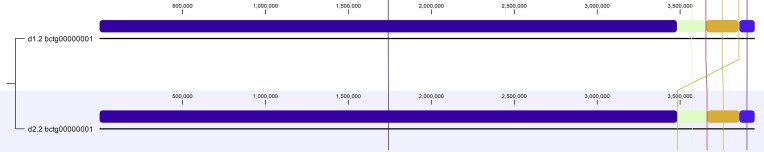
WGA of haplotigs d1.2 and d2.2 for chromosome 2 of *Y. lipolytica* YB-423 (ATCC 18942).

**Figure 4:**
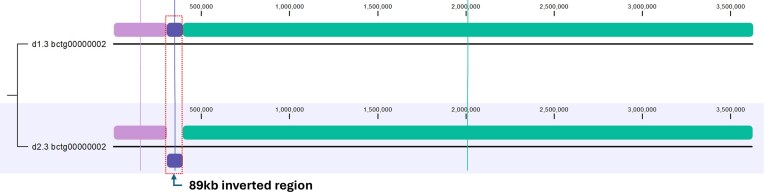
WGA of haplotigs d1.3 and d2.3 for chromosome 3 of *Y. lipolytica* YB-423 (ATCC 18942).

**Figure 5:**
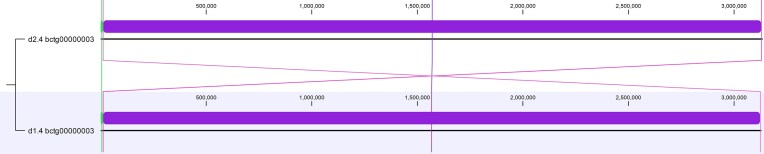
WGA of haplotigs d1.4 and d2.4 for chromosome 4 of *Y. lipolytica* YB-423 (ATCC 18942).

**Figure 6:**
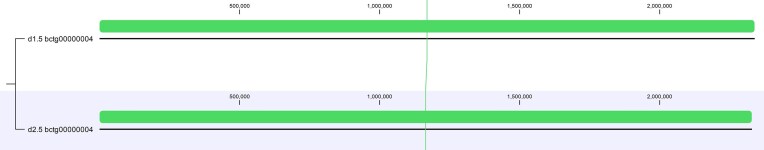
WGA of haplotigs d1.5 and d2.5 for chromosome 5 of *Y. lipolytica* YB-423 (=ATCC 18942).

**Figure 7:**
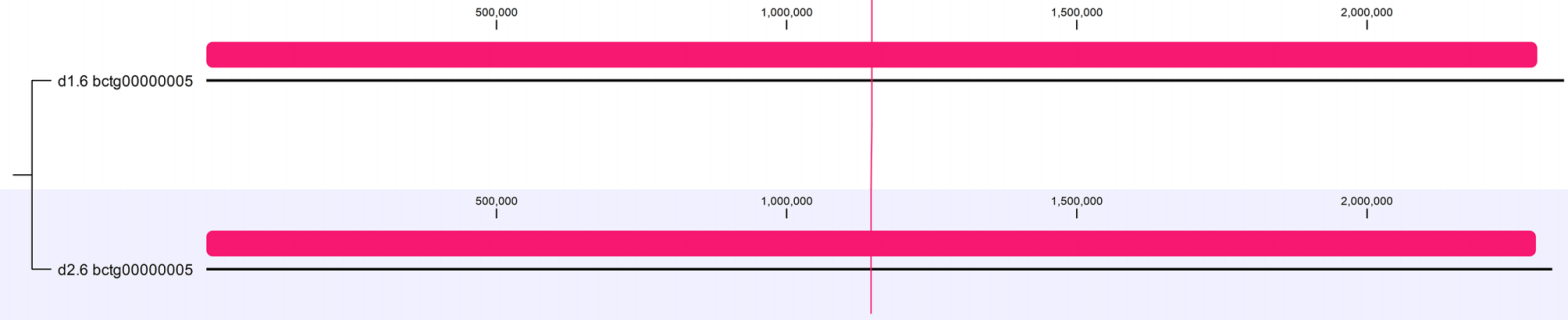
WGA for haplotigs d1.6 and d2.6 for chromosome 6 of *Y. lipolytica* YB-423 (=ATCC 18942).

**Figure 8:**
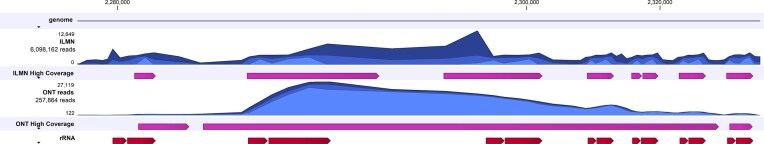
3’ Terminal end of chromosome 6 (haplotig d1.6) showing seven tandem repeats of rDNA loci and statistically significant high-coverage regions for both ILMN and ONT reads (histograms).

The haplotigs for chromosome 2 (haplotigs d1.2 and d2.2, Figure 3) featured a continuous 3.5 Mb aligned sequence (dark blue LCB), disrupted only by a 6.5 kb inverted transposon and a smaller 600 bp inversion. Chromosome 3 haplotigs (d1.3 and d2.3, Figure 4) were found to have a large 89 kb inversion harboring 28 protein-coding genes as well as a small 714 bp Ylt1 retrotransposon unique to d2. Chromosome 4 (d1.4 and d2.4, Figure 5) shared a contiguous 3.1 Mb sequence (purple LCB) flanked by reciprocal inverted regions near the terminal ends of the chromosome. The haplotig alignments for chromosome 5 (Figure 6) and chromosome 6 (Figure 7) also exhibited high structural similarity consisting of a single homologous LCB spanning their entire lengths. Chromosome 6 haplotigs differed by over ~20 kb in length, however, likely reflecting limitations in resolving repetitive rDNA gene clusters at their terminal ends (see below and Figure 8).

Overall, structural variations and differences in the lengths of each haplotig pair consistently correlated with an uneven distribution of retrotransposons and their relics found throughout the genome (see below). These were consistently supported by ONT reads spanning the regions in one haplotig, but not in its match, further strengthening the likelihood that these differences are true biological variations rather than sequencing or assembly artifacts.

### Diploid assembly genome features

#### Ribosomal RNA genes

We identified multiple rDNA repeat units (25S, 5.8S, 18S) in tandem arrays at the terminal ends of chromosomes 1, 3, 4, and 6. The longest rDNA locus assembled included seven copies of rRNA genes spanning a 60 kb region at the end of chromosome 6. This region was identified, however, as having significant coverage of both ONT (27,119×, *p* = 4.4eE-09) and ILMN (12,649×, *p* = 1.8E-08) reads, strongly suggesting that seven copies of rDNA loci is a significant underestimation of the true number found in this region (Figure 8). A similar jump in read-depth was observed at all other rDNA loci in the genome as well (data not shown). In contrast to rDNA loci being detected on chromosomal ends, additional RNA genes were found scattered throughout the genome, including 932 tRNA, 237 5S rRNA, 78 snRNA, and 31 other ncRNA genes.

#### Tandem repeats

The diploid genome of the type-strain was found to have an estimated 12,635 tandem repeat regions (TRRs), comprising 941 MB of genome sequence (~2.3% of the genome). One set of sister haplotigs was found to have 889 more TRRs than the other (6,762 vs. 5,873), but this is likely an artifact of the sorting method used for haplotigs in the final assembly by *necat*. Due to our minimum score required for identification, the smallest TRR identified was a ~30 bp TATA box binding site. Interestingly, the longest TRR was a 17,551 bp intergenic region in d1.2 composed of 30 copies of a smaller 594 bp repetitive elements that share >95% identity. This region shares a large 32 kb intergenic region with four other TRRs of 45 bp, 85 bp, 93 bp, and 3.5 kb, respectively (Figure 13). The same allelic region was also found in d2.2. The homologous region in W29 (based on the synteny of flanking genes) is homologous to YALI1_C08298g, a partial mRNA pseudo gene. The number of TRR’s found in each haplotig is show in Table 1.

#### Homopolymer regions

Due to the deep long-read sequencing used to produce the type strain genome assembly, we were able to identify and resolve 58 homopolymer regions in the genome, the longest of which being 116 bp in length. These included 56 polyA tracks (28 in each haplotig set) and two 36 bp polyG tracks. Interestingly, two of these polyA-rich regions (in each haplotig set) were found in putative genes with similarity (determined by BLAST) to YALI1_A05168g (98% identity) and YALI1_D16990g (96% identity), respectively. The former homopolymer is found on the sense strand of the extended 5’ UTR for YALI1_D16990g and expected to affect the putative protein sequence. The latter homopolymer, however, is found (anti-sense) in the putative protein coding region of a YALI1_D16990g homolog in an unusual low-complexity 60 bp polyU-rich sequence. This sequence is predicted to code for a degenerate series of Phe rich motifs not found in any other known protein (based on BLAST search). This suggests that YALI1_D16990g (and the protein AOW04031, which has a stretch of 20xPhe residues) is an artifact in the W29 genome that was erroneously propagated to the type strain genome.

#### Retrotransposon analysis

We identified 377 retrotransposon sequences in the diploid assembly, ranging from 35 bp transposon relics sequences to 7.5 kb full length Ylli transposons. We refined our annotation of transposons by manually obtaining all transposon element sequences found in all pre-existing *Y. lipolytica* genome assemblies in NCBI and the ATCC Genome Portal (Nguyen et al., 2024). Next, we clustered these sequences using an alignment-free k-mer spectra approach, and then serially aligned clusters using Clustal W. Consensus sequences from each aligned cluster were then used to iteratively search against the diploid assembly using BLAST (with default settings) to identify all full-length transposons and their relics that may be present in the assembly. These results revealed 12 potentially functional Ylli transposons and 1 Tyl6 transposon as well as an estimated 364 transposon relics scattered throughout the genome (Table 2). Further studies are required to determine the degree of functionality of these endogenous transposons in the type strain.

**Table 2:** Retrotransposon diversity.

Transposon type	Count
Fotyl relic	2
LTRyl1 relic	125
LTRyl3 relic	12
LTRyl6 relic	5
LTRyl7 relic	40
LTRyl8 relic	14
Mutyl relic	6
Tyl6	1
Tyl6 relic	1
Ylli	12
Ylli relic	150
Undetermined	9

### Comparison to CLIB89/W29

#### Genetic differences

Since the NCBI reference assembly for the species is for W29, we sought to use it for comparative genomics and variant analysis. To do this, we mapped 128 M ILMN reads (obtained from the type strain assembly) to the W29 assembly using CLC. After removing duplicate mapped reads, we next called variants using CLC’s implementation of *lofreq* (Wilm et al., 2012) and retained only high-quality variants (*p* < 0.01, see Methods). After filtering, a total of 30,209 heterozygous and 38,827 homozygous variants were found, including 58,974 SNVs, 1,714 MNVs, 4,839 deletions, 3,236 insertions, and 273 replacements. Furthermore, a total of 6,470 nonsynonymous variants were found in 1,654 protein coding genes. We also carried out GSEA (as described above for the haploid consensus assembly), and found a significant enrichment of variants in genes associated with transmembrane transport (GO:0055085, p 6.54E-13 237 genes out of 302), a similar finding to the results found for the type strain itself after analyzing the haploid consensus assembly—further emphasizing genetic diversity found among strains of the species in this biological function. A complete list of variants and the results of the GSEA are shown in Tables S4 and S5.

#### Structural differences

**Figure 9:**
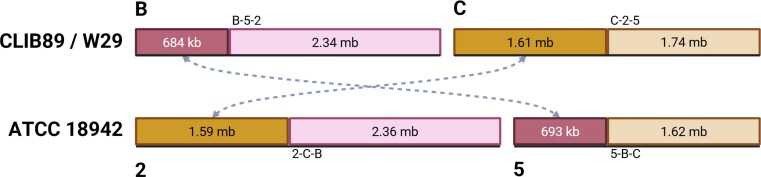
RTT of W29 chromosomes B & C, relative to the type-strain’s chromosomes 2 and 5. Approximate segment lengths shown in kb or mb, and four translocation junctions are uniquely labeled (B-5–2, C-2–5, 2-C-B, and 5-B-C). Type-strain chromosomes 2 and 5 from only one of the two haplotig sets is shown, as the structure is identical for each set.

**Figure 10:**
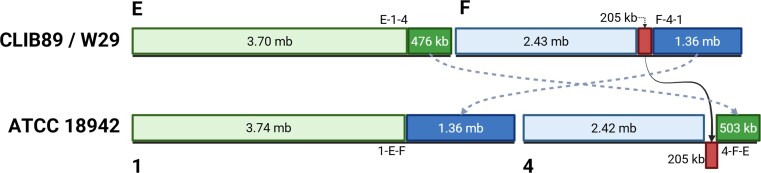
RTT of W29 chromosomes E & F relative to the type strain’s chromosomes 1 and 4. Approximate segment lengths shown in kb or mb, and four translocation junctions are uniquely labeled E-1–4, F-4–1, 1-E-F, and 4-F-E). A 205 kb inverted repeat region is indicated. Type strain chromosomes 2 and 5 from only one of the two haplotig sets is shown as the structure is identical for each set.

The current RefSeq assembly is GCA_001 761 485 for *Y. lipolytica* CLIB89/W29 (Magnan et al., 2016). For structural comparison purposes, we split our diploid assembly of the type strain into two independent sets of haplotigs and conducted WGA against the W29 assembly to better understand potential structural differences between these two strains. The relative assignment of the type strain haplotigs to W29 chromosomes is listed in Table 1. The results of the WGA show significant differences in gross genome structure between the two strains (Figure 1). While W29 chromosomes A and D had the same overall structure as their homologs in the type strain genome, the other chromosomes had significant reciprocal terminal translocations (RTT), the largest being 1.36 mb in length relative to the genome of the type strain. The RTTs for W29 chromosomes B & C are shown in Figure 9, and RTTs for E & F are shown in Figure 10.

The RTT in W29 C-2–5 (Figure 9) occurs in the middle of the hypothetical protein coding gene YALI1_C16133g. In YB-423, this junction introduces a premature stop codon in a cryptic sequence for the gene (2-C-B, Figure 9). No transposon or other relic sequences for mobile elements were found within 1 kb. To ensure this RTT was not the result of an assembly artifact, we manually inspected the ONT and ILMN data mapped to this RTT junction and found deep high quality ILMN reads (*Q* > 30,741×) and ONT reads (431×) spanning this locus, supporting the assertion that this RTT is indeed present in the type-strain genome. The junction for the reciprocal RTT (5-B-C) occurs 172 nt downstream of a gene nearly identical to W29’s YALI1_B06839g. Similarly, no cryptic mobile elements or transposon relics were found within 1 kb, and inspection of the NGS data mapped to this locus revealed deep coverage of both ILMN (549×) and ONT (458×) reads spanning this locus. The RTT junction for E-1–4 in W29 is in an intergenic region 341 nt downstream from YALI1_E36984g (Figure 10). Like the RTT’s described above, the reciprocal RTT junction in YB-423 (1-E-F) was not found to be adjacent to any transposon relics or cryptic LINE elements, and the NGS data strongly supports the 1-E-F junction with a coverage of 360× ILMN and 449× ONT reads spanning the locus.

**Figure 11:**
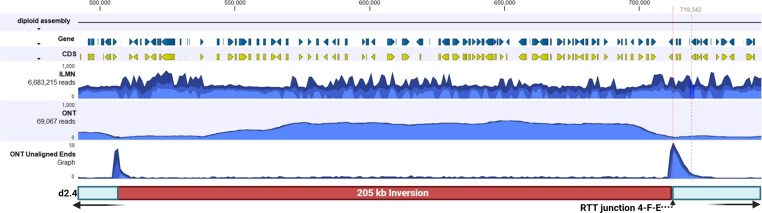
205 kb region inverted in the genome of YB-423 (ATCC 18942). Terminal 3’ end is marked with the 2-F-E RTT junction. Tracks of this region show genes, CDS regions, ILMN and ONT reads coverage, and the peaks for unaligned ends of ONT reads.

**Figure 12:**
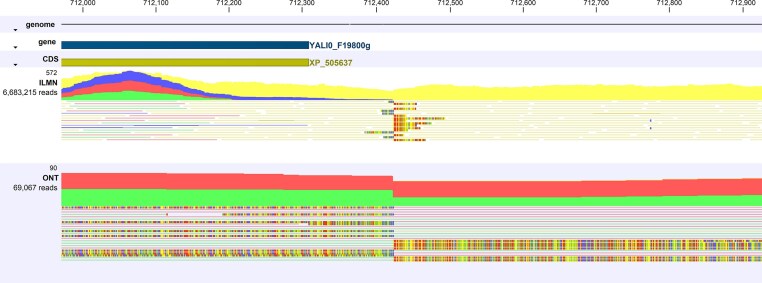
Nucleotide resolution view of the RTT junction 2-F-E highlighting the unaligned ILMN and ONT reads spanning this locus.

Compared to the W29 assembly, the reciprocal RTT junction F-4–1 falls at the end of a large inverted 204 kb region in YB-423 (Figure 10). Inspection of the remapped NGS reads at this region in the diploid assembly revealed a considerable number with unaligned ends at the boundaries of the region (Figure 11). Close inspection of the boundaries of this region confirmed a sharp, heterozygous breakpoint at both the 5’ and 3’ terminal ends of the region that exactly matched the same position of the RTT boundaries for the 4-F-E RTT (Figure 12 ). Furthermore, it is notable that the coverage of ONT reads spanning this region was nearly doubled, suggesting this region may be a potentially large, heterozygous inverted repeat region.

**Figure 13:**
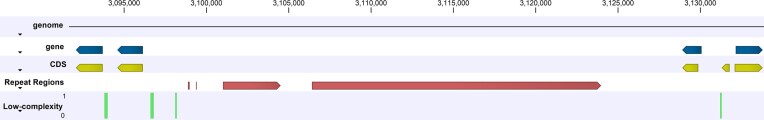
32 kb intergenic region in d1.2 carrying the largest terminal repeat region (17.5 kb) of 30 repeating 562 bp repeat elements. Four other, smaller, TRRs are found just upstream of the same region.

Overall, the structure of YB-423 is substantially divergent from W29, not only at the level of individual SNPs (as expected), but also at level of the structure of the overall genome.

## Discussion

This study presents the first high-quality, fully phased diploid genome assembly for *Y. lipolytica* YB-423 (=ATCC 18942), the type-strain for the species. A summary of general features for the diploid assembly is provided in Table S6. This work significantly advances our understanding of the type strain’s sequence and genome structure, but importantly it has also illuminated the extensive degree of allelic and structural diversity within the diploid genome present in wild-type *Y. lipolytica*. While prior draft haploid consensus assemblies available from NCBI and the ATCC Genome Portal provided a foundation for the genetic sequence of the type strain, the phased diploid assembly presented here enables the resolution of haplotype-specific allelic variants within their proper genomic and structural contexts. A phased diploid genome constructed using both long- and short-read sequencing technologies reveals a previously obscured, but extensive, degree of heterozygosity masked in prior assemblies. The diploid assembly identified 13,908 high-confidence heterozygous alleles, including 1,062 non-synonymous alleles that may have an influence on phenotype. GSEA highlighted significant enrichment of variants within genes involved in transmembrane transport for both the type strain and (by comparison) the NCBI RefSeq reference assembly for W29, providing an intriguing hypothesis related to *Y. lipolytica’s* versatile metabolic capabilities and range. Structural comparisons further identified numerous heterozygous structural diversities including inversions, transpositions, and retrotransposons, illustrating significant intragenomic genomic variability. While the extensive heterozygosity and structural variation in YB-423 suggest historical recombination and retention of allelic diversity, we emphasize that we cannot infer recent clonal expansion or population-level diversity from this single genome assembly. Nonetheless, the features we have revealed in the type strain are expected to reflect the natural genetic variability of wild-type *Y. lipolytica*, but confirmation would require broader population sampling. Such investigations would require a larger comparative genomics study across multiple diploid and haploid strains, coupled with a clear lineage map of their genetic relatedness. These insights provide a robust genomic framework essential for future comparative and functional genomic studies. The diploid reference assembly for *Y. lipolytica* presented above will serve to enable improved downstream genetic modifications for industrial microbiology applications and a better understanding of natural genetic variability, facilitating the rational design of optimized *Y. lipolytica* strains capable of efficiently metabolizing diverse substrates.

## Supplementary Material

kuag002_Supplemental_Files

## Data Availability

The annotated haploid consensus and phased diploid assemblies for *Y. lipolytica* YB-423 (=ATCC 18942) have been deposited into the ATCC Genome Portal (https://genomes.atcc.org). The haploid consensus assembly and raw sequencing data have also been deposited to NCBI and can be found in BioProject PRJNA1290264.
